# Multi-Omics Reveal Antioxidant Effects of Bardoxolone Methyl in the Phase 2 Study of Bardoxolone Methyl in Patients with CKD and Type 2 Diabetes Study

**DOI:** 10.34067/KID.0000000853

**Published:** 2025-06-11

**Authors:** Kentaro Yoshioka, Hiroto Kaneko, Waka Haruyama, Tetsuro Tomiyama, Atsuko Takami, Tetsuya Kitayama, Kohei Yamasaki

**Affiliations:** 1 Research Division, Kyowa Kirin Co., Ltd., Tokyo, Japan; 2 Development Division, Kyowa Kirin Co., Ltd., Tokyo, Japan

**Keywords:** CKD, diabetic nephropathy, oxidative stress, metabolomics, proteomics

## Abstract

**Key Points:**

- The study aims to clarify the effects of bardoxolone methyl using proteomics and metabolomics analyses of plasma and urine from The Phase 2 Study of Bardoxolone Methyl in Patients with CKD and Type 2 Diabetes.
- This study demonstrated the first evidence in humans that bardoxolone methyl activates an antioxidant response.

**Background:**

NF erythroid 2-related factor 2 (NRF2) is crucial for defense against oxidative stress. In The Phase 2 Study of Bardoxolone Methyl in Patients with CKD and Type 2 Diabetes, bardoxolone methyl, an NRF2 activator, was shown to increase the GFR in patients with diabetic kidney disease. Although nonclinical reports suggest that bardoxolone methyl acts mainly through NRF2-mediated antioxidant response activation, this has not been proven in clinical settings. This study assessed its effects using plasma and urine from The Phase 2 Study of Bardoxolone Methyl in Patients with CKD and Type 2 Diabetes.

**Methods:**

Patients received either bardoxolone methyl or placebo daily for 16 weeks. Urine and plasma samples were collected at baseline, after 16 weeks of dosing, and 4 weeks postdosing and were cryopreserved for an analysis. A total of 45 patients in the bardoxolone methyl group and 52 in the placebo group were subjected to proteomic and metabolic analyses. Proteomic profiling was conducted using the SOMAscan assay, whereas metabolomics analyses were performed by Human Metabolome Technologies, Inc.

**Results:**

Plasma proteomics revealed that two oxidative stress–related pathways have been significantly changed by bardoxolone methyl treatment. Among these two pathways, antioxidative proteins and proteins that enhance the antioxidative process were significantly increased. Some of these increased proteins were known to be NRF2 target proteins. Similarly, urine proteomics revealed that four pathways were changed, and antioxidative proteins that are the target proteins of NRF2 were increased. Furthermore, seven metabolites that potentiate the antioxidative effect were significantly increased in urine.

**Conclusions:**

This study demonstrated the first evidence in humans that bardoxolone methyl activates an antioxidant response.

## Visual Abstract

**Figure:**
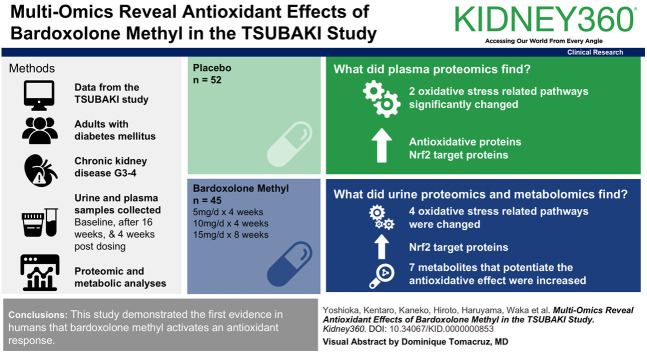

## Introduction

The respiratory system continuously acquires oxygen to produce energy *via* aerobic respiration in mitochondria.^1^ While this system is necessary for the function of all living cells, it produces reactive oxygen species (ROS) as a byproduct, which are harmful to living cells due to their high reactivity and are involved in a number of diseases.^2–7^

NF erythroid 2–related factor 2 (NRF2) is the intracellular master regulator of antioxidative stress genes, playing a key role in defending against ROS, because it is activated by sensing oxidative stress.^3–6^ Under normal cellular conditions, NRF2 is steadily captured by Kelch-like ECH-associated protein 1 (KEAP1), a subunit of E3 ubiquitin-ligase specific for NRF2, and then degraded by ubiquitin–proteosome system.^4^ By contrast, in cells under oxidative stress, NRF2 is activated through detaching from KEAP1 because a conformation change of KEAP1 is induced by the binding of ROS to specific cysteine residues.^8^ Recent study suggests that transcriptional activation of NRF2 target genes is achieved by *de novo* synthesis of NRF2, rather than by the release of NRF2 from the KEAP1 complex.^9^

Several reports have indicated the relationship between the KEAP1-NRF2 system and kidney disease. For instance, NRF2 null mice exhibited exacerbation of lupus-like nephritis.^10^ Similarly, streptozotocin-induced nephropathy was found to be exacerbated in NRF2 null mice.^11–13^ Furthermore, mitigation of proximal tubular injury in an ischemic/reperfusion model^14^ and amelioration of tubular fibrosis in a unilateral ureteral obstruction model were observed in KEAP2 hypo mice which have hypomorphic alleles in KEAP1.^15^ Bardoxolone methyl, a semisynthetic triterpenoid compound, can potently activate NRF2.^16,17^ A *post hoc* analysis of the BEACON trial, which is a phase 3 study of patients with diabetic kidney disease (DKD), revealed that the eGFR remained above the baseline on treatment with bardoxolone methyl.^18^ In addition, in The Phase 2 Study of Bardoxolone Methyl in Patients with CKD and Type 2 Diabetes (TSUBAKI), which is a Japanese phase 2 trial of patients with stage G3 and G4 CKD, bardoxolone methyl increased the measured GFR in the stage G3 group from the baseline at week 16, as determined using inulin clearance.^19^ As oxidative stress is reportedly involved in kidney disease progression,^20,21^ the antioxidant effect of bardoxolone methyl is considered to have contributed to this outcome. However, direct evidence supporting the antioxidant effect in clinical trial induced by bardoxolone methyl has not been presented.

We conducted this study to obtain clear evidence regarding the antioxidant effect exerted by bardoxolone methyl using proteomics and metabolomics analyses of plasma and urine derived from patients in the TSUBAKI study. This is the first report to detect the NRF2-mediated upregulation of the antioxidant effect in patients with CKD by bardoxolone methyl treatment.

## Methods

### Overview of the TSUBAKI Study and Sample Collection

All of the samples in this study were obtained in the reported clinical trial of the TSUBAKI study, the protocol and patient background information of which have already been reported.^19^ In brief, TSUBAKI (ClinicalTrials.gov: NCT02316821) was a randomized, multicenter, double-blind, placebo-controlled trial study that enrolled patients with type 2 diabetes and stage G3–4 CKD. Patients received bardoxolone methyl or placebo orally once daily for 16 weeks. The starting dose was 5 mg/d, followed by dose escalation, as tolerated, to 10 mg/d at week 4 and 15 mg/d at week 8. Urine and EDTA-plasma of the patients were collected at baseline, 16 weeks from the beginning of dosing, and 4 weeks after the end of dosing. These samples had been cryopreserved until the analysis. The samples collected at three time points as planned from 45 patients for the bardoxolone methyl dosing group and 52 for the placebo group were all tested for proteomic and metabolic analyses. All of the patients gave their signed consent agreeing to the use of their samples in this research. Analyses were performed according to the protocol approved by the Ethics Committee of Kyowa Kirin Co., Ltd. (Approved number: 2018_003) and adhered to the Declaration of Helsinki. Patients were given the opportunity to exclude themselves from the substudy *via* an opt-out approach at study initiation.

### Proteomics Analyses

Plasma and urine proteomic profiling was performed using the SOMAscan^22^ platform based at the SomaLogic Laboratory (Boulder, CO). The SOMAscan is a multiplexed proteomics platform that uses DNA aptamers (single-stranded DNA molecules) selected to bind specific protein targets. The bound proteins are then quantified. The Human Plasma SOMAscan 7k kit with a set of calibration and normalization samples was used following the manufacturer's recommended protocol. The SOMAscan results are quantified on a hybridization microarray and reported in relative fluorescent units. Data standardization and calibration were performed according to the SOMAscan platform data quality control protocols. The SOMAscan data passed quality control criteria, otherwise data were excluded from the analysis. In cases where data were obtained by several aptamers for a single protein, data with the highest mean signal were used. Urine data were normalized by creatinine levels obtained by metabolomics analyses.

### Metabolomics Analyses

Metabolomics analyses were performed by Human Metabolome Technologies, Inc. (HMT, Tokyo, Japan). They analyzed plasma and urine samples by liquid chromatography time-of-flight mass spectrometry and capillary electrophoresis time-of-flight mass spectrometry (CE-TOF-MS) using HMT Advanced Scan methods, respectively.^23^ Liquid chromatography time-of-flight mass spectrometry targeted lipophilic metabolites in plasma samples, and CE-TOF-MS targeted water-soluble metabolites in urine samples.

The plasma metabolomics is performed as the following procedure. Each 100 *μ*l of plasma sample was mixed with 300 *μ*l of 0.1% formic acid in methanol (*v/v*) containing internal standards and centrifuged (9100×*g*, 4°C, 10 minutes). 250 *μ*l of supernatant and 550 *μ*l of 0.1% formic acid in water were mixed and filtrated at 4°C by using solid phase extraction column (MonoSpinC18, 5010-2170, GL Sciences Inc., Tokyo, Japan). The filtrate was then purified with 0.1% formic acid solution and 0.1% formic acid–25% methanol solution. Subsequently, the purified lipid metabolites were dissolved with 200 *μ*l of 0.1% formic acid in methanol. The compounds were measured in the positive and negative modes of liquid chromatography/mass spectrometry–based metabolome analysis. Peaks detected in the liquid chromatography/mass spectrometry analysis were extracted using automatic integration software (MultiQuant, AB Sciex) to obtain peak information, which includes m/z, retention time, and peak area. The peak area was then converted to relative peak area by the following equation:

Relative Peak Area=Metabolite Peak Area/(Internal Standard Peak Area×Sample Amount).

Putative metabolites were then assigned from HMT's standard library on the basis of m/z and migration time (MT).

The urine sample metabolomics is performed as the following procedure. Each 20 *μ*l of urine sample were mixed with 20 *μ*l of Milli-Q water containing internal standards (100 *μ*mol/L) and 60 *μ*l of Milli-Q water. The mixture was then filtrated at 4°C through 5-kDa cutoff filter (ULTRAFREE-MC-PLHCC, Human Metabolome Technologies, Yamagata, Japan) to remove macromolecules. The compounds were measured in the cation and anion modes of CE-TOF-MS–based metabolome analysis. The samples were diluted for the measurement, to improve analysis qualities of the CE-MS analysis. Peaks detected in the CE-MS analysis were extracted using automatic integration software (MasterHands ver. 2.19.0.2 developed at Keio University) to obtain peak information, which includes m/z, MT, and peak area. The peak area was then converted to relative peak area by the following equation:

Relative Peak Area (Cation mode)=Metabolite Peak Area/Creatinine Peak Area×1000;

Relative Peak Area (Anion mode)=(Metabolite Peak Area/Internal Standard Peak Area of Anion mode)×1000/(Creatinine Peak Area/Internal Standard Peak Area in Cation mode).

Putative metabolites were then assigned from HMT's standard library on the basis of m/z and MT.

### Statistical Analyses of Proteome Data

The log_2_-fold change (Log_2_FC) of protein expressions at week 16 compared with baseline was calculated. In brief, a positive Log_2_FC value means an increase in the expression, whereas a negative value means a decrease. Differentially expressed proteins (DEPs) were defined as the proteins having |mean Log_2_FC| >0.58 among each group (bardoxolone methyl/placebo). We then tested whether the DEPs were enriched in given inflammation-related and antioxidant-related pathways using the Fischer exact test. Nine pathways related to the inflammatory and antioxidant process referred from Gene Ontology, MetaCore (Clarivate plc, Jersey) and WikiPathways (as of December of 2022) were used for the pathway enrichment analyses (Supplemental Table 1). In addition, we tested whether Log_2_FC of each protein has different distribution by group (bardoxolone methyl/placebo) using the Wilcoxon rank sum test. This statistical test was performed for 246 proteins included in the nine pathways used for the pathway enrichment analysis and the inflammatory protein signature in diabetes.^24^

### Statistical Analyses of Metabolome

Analyzed metabolites were selected on the basis of the involvement with antioxidation and inflammation: 54 lipophilic metabolites in plasma samples and 39 water-soluble metabolites in urine samples. The Log_2_FC of metabolites at week 16 compared with baseline was calculated. We tested whether the Log_2_FC of each analyzed metabolite had different distributions by group (bardoxolone methyl/placebo) using the Wilcoxon rank sum test.

### Data Adaptation and Statistical Testing

Proteins/metabolites with two-thirds or more missing values at baseline and/or at week 16 in the bardoxolone methyl group and/or placebo group were excluded from the analysis. Missing values caused by being below the lowest limit of detection were imputed by half of the minimum detected value. Missing values caused by quality criteria of measurement were not imputed. The Fischer exact test and Wilcoxon rank sum test were performed using the function fisher.test of R (Version 4.2.1) and the R package coin (Version 1.4.2), respectively. All statistical significance assessments were performed with the two-sided test. The Benjamini–Hochberg procedure was performed to control the false discovery rate, and its criterion was set to 5%.

## Results

### Patient Demographics and Baseline Characteristics

Of the patients who participated in the TSUBAKI study (both cohorts G3 and G4),^19^ plasma and urine samples were collected and analyzed from a total of 97 patients (45 patients for the bardoxolone methyl dosing group and 52 patients for the placebo group). Bardoxolone methyl was administered to the patients in the dosing group using a titration scheme (see Methods), with the highest dose being 5 mg/d in one patient, 10 mg/d in five patients, and 15 mg/d in 39 patients. This population represented 81% (bardoxolone methyl, 69%; placebo, 95%) of the overall TSUBAKI study population.^19^ Note that patients who discontinued treatment before the end of the study were not included in this multi-omics study. In generally, demographics and baseline characteristics of patients with available samples were consistent with the overall TSUBAKI study population (Table 1). Because urine samples were not collected from one patient in the placebo group at baseline and at the period of 16 weeks, we performed proteomic and metabolomic analyses on 291 plasma and 289 urine samples. This patient was not included in the statistical analyses.

**Table 1:** Demographics and baseline characteristics of patients in The Phase 2 Study of Bardoxolone Methyl in Patients with CKD and Type 2 Diabetes with available multi-omics samples

Characteristic	Bardoxolone Methyl Group (*n*=45)	Placebo Group (*n*=52)^a^
**Sex, *No.* (%)**		
Female	15 (33.3)	15 (28.8)
Male	30 (66.7)	37 (71.2)
Age, yr, mean (SD)	68.0 (7.4)	69.5 (7.2)
Weight, kg, mean (SD)	64.53 (10.00)	69.01 (14.45)
BMI, kg/m^2^, mean (SD)	24.97 (3.44)	25.88 (4.14)
eGFR, ml/min per 1.73 m^2^, mean (SD)	40.78 (12.76)	40.79 (12.18)
**CKD stage by eGFR, *No.* (%)**		
Stage G3a	21 (46.7)	25 (48.1)
Stage G3b	9 (20.0)	13 (25.0)
Stage G4	15 (33.3)	14 (26.9)
Serum creatinine, mg/dl, mean (SD)	1.411 (0.548)	1.413 (0.543)
ACR, mg/g, mean (SD)	384.49 (595.33)	245.98 (469.81)
BP, mm Hg, mean (SD)		
Systolic	131.3 (16.2)	129.0 (16.7)
Diastolic	74.0 (10.3)	71.1 (10.1)
HbA_1c_, %, mean (SD)	6.92 (0.79)	7.18 (0.86)

ACR, albumin-to-creatinine ratio; BMI, body mass index; HbA_1c_, hemoglobin A_1c_.

a Urine samples for one patient in the placebo group at baseline and at 16 weeks were not collected, so this patient was excluded from the statistical analyses.

### Proteomics Analyses of Plasma and Urine Samples of Patients in TSUBAKI Study

**Figure 1:**
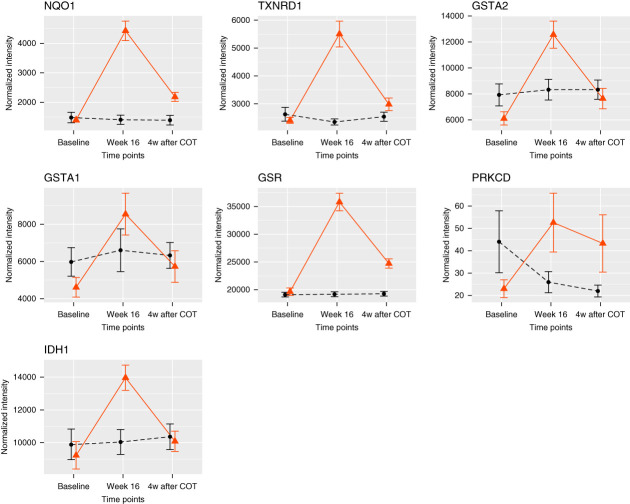
**The time course of proteins measured in plasma.** DEPs in DEP-enriched pathways for which the expression change in the bardoxolone methyl group was significant compared with the placebo group are shown. The vertical axis represents the normalized intensity of protein expression. Data are shown as the mean of each group±SEM. The bold red line represents the bardoxolone methyl group, and the black dotted line represents the placebo group. DEP, differentially expressed protein. See Supplemental Table 2 for long form of protein names. DEP, differentially expressed proteins.

With SOMAscan assay of plasma samples, 6363 proteins were detected without the exclusion because of missing values. As the primary pharmacology of bardoxolone methyl is considered to be its anti-inflammatory and antioxidant effects through NRF2 activation, this study focused on these mechanisms of action, and enrichment analyses of proteomic data were performed on the pathways shown in Supplemental Table 1. An enrichment analysis revealed that the glutathione (GSH) metabolic process and NRF2 regulation of oxidative stress response process were DEPs enriched in the bardoxolone methyl group (Table 2). Among these pathways, seven proteins were DEPs, and all of them were increased at 16 weeks compared with baseline (Supplemental Table 2). By contrast, no significant change was observed in the placebo group (Table 2). Furthermore, among DEP-enriched pathways in the bardoxolone methyl group, the increased expression of 15 proteins (including all seven DEPs) and the decreased expression of six proteins were statistically significant compared with the placebo group (Supplemental Table 2). The expression of proteins increased by treatment with bardoxolone methyl tended to be decreased with withdrawal of the treatment (Figure 1).

**Table 2:** A pathway enrichment analysis of differentially expressed proteins in plasma

Pathway	Adjusted *P* Value (Q) of Fisher Exact Test	
	Bardoxolone Methyl Group	Placebo Group
Glutathione metabolic process	0.0061^a^	1.0
Glutathione biosynthetic process	1.0	1.0
Inflammatory response pathway	1.0	1.0
Chemokine signaling pathway	0.55	1.0
Type 2 IFN signaling	0.50	1.0
Cytokines and inflammatory response	1.0	1.0
The role of the KEAP1/NRF2 pathway in skin sensitization	0.20	1.0
NRF2 regulation of oxidative stress response	0.0051^a^	1.0
Antiviral and anti-inflammatory effects of NRF2 on SARS-CoV-2 pathway	0.069	1.0

KEAP1, Kelch-like ECH-associated protein 1; NRF2, NF erythroid 2-related factor 2; SARS-CoV-2, severe acute respiratory syndrome coronavirus 2.

a Statistical analysis results with Q<0.05.

**Figure 2:**
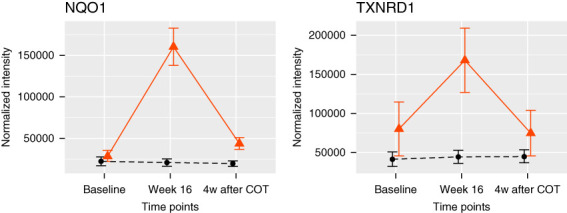
**The time course of proteins measured in urine.** DEPs in DEP-enriched pathways for which the expression change in the bardoxolone methyl group was significant compared with the placebo group are shown. The vertical axis represents the normalized intensity of protein expression. Data are shown as the mean of each group±SEM. The bold red line represents the bardoxolone methyl group, and the black dotted line represents the placebo group. See Supplemental Table 3 for long form of protein names.

Urine proteins were also subjected to a pathway analysis, as described above. A total of 4063 proteins were detected without the exclusion because of missing values. The treatment with bardoxolone methyl resulted in significant DEP enrichment of the following pathways: GSH metabolic process, GSH biosynthetic process, cytokine and inflammatory response, and NRF2 regulation of oxidative stress response (Table 3). Among these pathways, ten proteins were DEPs, and all of them were increased at 16 weeks compared with baseline (Supplemental Table 3). Furthermore, a statistical test comparing with placebo revealed the significant increase of two proteins (also DEPs) and decrease of one protein among DEP-enriched pathways in the bardoxolone methyl group (Supplemental Table 3). The expression of two significantly increased proteins peaked at week 16 and returned to near baseline by 4 weeks after drug withdrawal (Figure 2), just as in plasma.

**Table 3:** A pathway enrichment analysis of differentially expressed proteins in urine

Pathway	Adjusted *P* Value (Q) of Fisher Exact Test	
	Bardoxolone Methyl Group	Placebo Group
Glutathione metabolic process	0.013^a^	1.0
Glutathione biosynthetic process	0.025^a^	1.0
Inflammatory response pathway	0.61	1.0
Chemokine signaling pathway	0.81	1.0
Type 2 IFN signaling	1.0	1.0
Cytokines and inflammatory response	0.025^a^	1.0
The role of KEAP1/NRF2 pathway in skin sensitization	0.42	1.0
NRF2 regulation of oxidative stress response	0.025^a^	1.0
Antiviral and anti-inflammatory effects of NRF2 on SARS-CoV-2 pathway	0.15	1.0

KEAP1, Kelch-like ECH-associated protein 1; NRF2, NF erythroid 2-related factor 2; SARS-CoV-2, severe acute respiratory syndrome coronavirus 2.

a Statistical analysis results with Q<0.05.

### Metabolomics Analyses of Plasma and Urine Samples of Patients in the TSUBAKI Study

Next, metabolomics analyses of plasma and urine samples were performed to evaluate the effects of bardoxolone methyl from a more phenotypic perspective. We detected 361 lipophilic metabolites in plasma samples (see Methods). Of the 54 lipophilic metabolites of interest, six were detected without the exclusion because of missing values. The results showed a significant decrease in cortisone and a significant increase in lysophosphatidylcholine (LPC; 18:0) and lactosylceramide (d18:1/18:0) in the bardoxolone methyl group compared with the placebo group (Supplemental Table 4).

**Figure 3:**
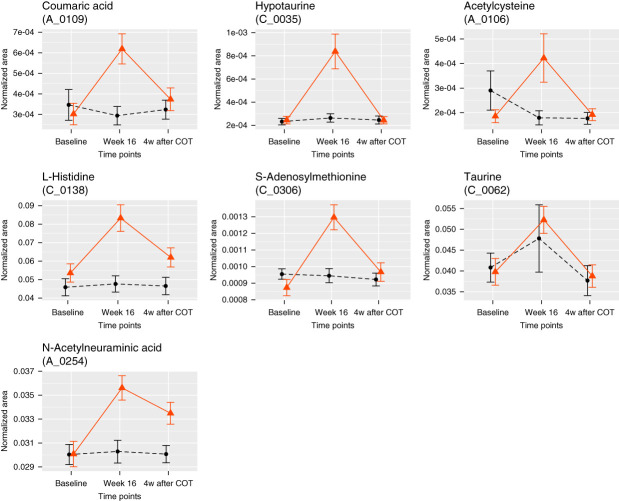
**The time course of urine content of water-soluble metabolites.** Metabolites for which the urine content change in the bardoxolone methyl group was significant compared with that in the placebo group are shown. The vertical axis represents the normalized area of metabolites. Data are shown as the mean of each group±SEM. The bold red line represents the bardoxolone methyl group, and the black dotted line represents the placebo group. One peak (A_0084) was also detected as significantly changed, but this peak is omitted in this figure because it is related to the mixture of metabolites.

We detected 594 water-soluble metabolites in urine samples (see Methods). Of the 39 water-soluble metabolites of interest, 24 peaks related to 20 metabolites were detected without the exclusion because of missing values. As shown in Supplemental Table 5, urine contents of seven metabolites—coumaric acid, hypotaurine, acetylcysteine, histidine, *S*-adenosylmethionine, taurine, and *N*-acetylneuraminic acid—were significantly changed by bardoxolone methyl treatment, and all were increased from the baseline. In addition to the peaks of these seven increased metabolites, one peak (A_0084) was also detected as significantly increased, but this peak was related to the mixture of metabolites. The urine contents of all the seven metabolites increased to the peak value at week 16 and returned to the baseline level by drug withdrawal (Figure 3).

## Discussion

Our multi-omics study showed that the treatment of bardoxolone methyl in patients with DKD activates NRF2-related antioxidative stress. Among the proteins increased from baseline to week 16 with bardoxolone methyl treatment (Figures 1 and 2 and Supplemental Tables 2 and 3), NQO1, TXNRD1, GSTA2, GSTA1, GSR, and IDH1 are direct NRF2-targeted proteins.^25,26^ Furthermore, GSS,^27^ GSTO1,^28^ and GSTM3,^26^ the values of which were significantly increased compared with the placebo group in plasma samples (Supplemental Table 2), are known downstream target proteins of NRF2.

Both proteomics and metabolomics suggest that the antioxidant effect was induced by the treatment of bardoxolone methyl. GSTA1, GSTA2,^26^ and NQO1^26,29^ are reported to be antioxidant proteins. CREBBP,^30^ PRKCD and PRKCG,^31^ which were significantly increased in plasma samples (Supplemental Table 2), are involved in NRF2 activation. IDH1 contributes to the production of NADPH, which is required for reduced GSH.^32^ TXNRD1 plays a role in maintaining the antioxidant system of thioredoxin by reducing the thioredoxin content.^29,33^ GSR, GSS, GSTO1, and GSTM3 play roles in the production and maintenance of the glutathione-based antioxidant system.^26,34,35^ MAFG, which is another protein increased in plasma samples (Supplemental Table 2), forms a complex with NRF2 to enhance the NRF2-mediated antioxidant effect.^36^ In addition, seven water-soluble metabolites—coumaric acid,^37,38^ hypotaurine,^39,40^ acetylcysteine,^41^ histidine,^42^ *S*-adenosylmethionine,^43,44^ taurine,^39,40^ and *N*-acetylneuraminic acid^45,46^—that were significantly increased in the urine of the bardoxolone methyl group are reported to exert antioxidant effects (Supplemental Table 5). Furthermore, as shown in Figures 1–3, the protein and metabolite values increased to a peak at week 16 and then returned to the baseline level after drug withdrawal. This recovery indicates that the antioxidant effects induced by treatment with bardoxolone methyl were reversible. On the basis of these findings, we conclude that an intrinsic antioxidant effect was activated by treatment with bardoxolone methyl.

However, interpreting the changes in plasma lipophilic metabolites remains challenging. The plasma metabolome showed significant changes in the cortisone, LPC (18:0), and lactosylceramide (d18:1/18:0) levels in the treatment group (Supplemental Table 4). Cortisone is an inactive form of the glucocorticoid hormone, and 11*β* hydroxysteroid dehydrogenase type 1 synthesizes the activated form, cortisol.^47^ The relationship between NRF2 and glucocorticoid hormones has not been studied extensively. The function of LPC (18:0) is complex, with some reports describing anti-inflammatory effects and others the opposite.^48^ Lactosylceramide (d18:1/18:0) has been reported to induce oxidative stress, whereas others report that it inhibits the activation of invariant natural killer T cells.^49^ The plasma metabolome data detected quantitatively in this study were limited to six of the 38 metabolites selected as candidates, making it difficult to comprehensively interpret the antioxidant effects of bardoxolone methyl.

Several NRF2 activators have been reported and are expected to become new therapeutic drugs because the physiological role of NRF2 is robust.^25^ The challenge is determining the pharmacodynamics in patients.^50^ Liu *et al.* attempted to detect NRF2 target gene induction in peripheral blood mononuclear cells derived from patients with autism spectrum syndrome after treatment with the NRF2 activator, sulforaphane.^51^ However, significant induction was difficult to observe. Zimmerman *et al.* analyzed NRF2 downstream gene alteration in peripheral blood mononuclear cells with enlarged cohort, but no significant induction of NQO-1 or HO-1 was detected.^52^ Hammer *et al.* detected temporary NQO-1 induction, but it had disappeared by 3 months.^53^ In this study, conversely, we were able to detect the significant induction of NRF2 target proteins, even at 16 weeks from the initiation of drug administration. In addition, this result was obtained using the samples from a phase 2 clinical trial with about 50 patients in each group, so we considered this evidence to be robust. The procedure of this study seems acceptable for use in a clinical trial because it measures the protein level in plasma noninvasively. Given the above, this result is sure to have a strong influence on both NRF2 research and subsequent clinical trials involving NRF2 activators because we provided direct evidence of the antioxidant effect of bardoxolone methyl in a controlled clinical trial.

In this study, KEAP1 in the plasma was increased (Supplemental Table 2), just as reported by Hammer *et al*.^53^ This result is attributed to the negative feedback of NRF2 activation. An increase in MAFG, which forms a heterdimer with NRF2 and activates transcription, was observed in this study. This result might indicate a new mechanism of self-activation of NRF2. These findings have prompted questions sure to lead to further research concerning NRF2.

We were unable to detect the anti-inflammatory effect of bardoxolone methyl for several reasons. First, owing to the lack of a healthy control, whether patients with DKD were in an inflammatory state that could be suppressed is unclear. Second, most of the proinflammatory cytokines were below the detectable level, making an evaluation difficult. In this study, the level of the chemokine CXCL1 in urine was decreased by bardoxolone methyl (Supplemental Table 3), but this change was detected at only the single-molecule level, and the pathway itself was unchanged. Therefore, we cannot conclude that this was caused by an anti-inflammatory effect.

Several limitations associated with this study warrant mention. First, the plasma protein source of the organs could not be determined. Conversely, urine proteins were thought to be released from the kidney, more specifically tubular or kidney blood vessels, because antioxidant proteins cannot penetrate the glomerular barrier due to their mol wt. We also did not perform a validation study. To verify the results, an analysis of samples from the validation cohort study in a similar-or-larger-scale trial, such as the AYAME study^54^ (ClinicalTrials.gov: NCT03550443), will be required. In the proteomics analysis, expressions in some proteins were highly variable even at baseline. For example, the coefficient of variation (CV, ratio of the SD to the mean) of PRKCD expressions in the whole analyzed population was 215% at baseline (Figure 1). The effect of bardoxolone methyl treatment on the expression of these proteins should be interpreted with caution, although a method robust to outlier values (Wilcoxon rank sum test) was used to test the difference in Log_2_FC between the two groups (bardoxolone methyl/placebo). The influence of proteins with high CV at baseline on the results of pathway analyses is considered as marginal because of their limited number. The number of proteins with more than 200% in the baseline CV was 566 of 6363 (8.9%) in plasma and 573 of 4063 (14.1%) in urine (Supplemental Figure 1).

This study demonstrated the first evidence in humans that bardoxolone methyl can induce an NRF2 activation-mediated intrinsic antioxidant effect. Because no previous report has shown this effect using plasma and urine obtained from controlled clinical trials, this study result is expected to strongly influence NRF2 clinical research.

## Supplementary Material

## Data Availability

Data related to transcriptomic, proteomic, or metabolomic data. Clinical Trial Data. Other. The datasets generated and/or analyzed during the current study are not publicly available as there are no public repositories for this type of dataset. The data are available from the corresponding author on reasonable request.

## Supplemental Material

Supplemental Table 1. Analyzed pathways in proteomics.

Supplemental Table 2. Proteins in the pathways modulated by bardoxolone methyl revealed by plasma proteomics. |Mean Log2FC| >0.58 are shown in bold font. Statistical analysis results with Q<0.05 are shown in bold font.

Supplemental Table 3. Proteins in the pathways modulated by bardoxolone methyl revealed by urine proteomics. |Mean Log2FC| >0.58 are shown in bold font. Statistical analysis results with Q<0.05 are shown in bold font.

Supplemental Table 4. Plasma lipophilic metabolites. Statistical analysis results with Q<0.05 are shown in bold font.

Supplemental Table 5. Urine water-soluble metabolites. Statistical analysis results with Q<0.05 are shown in bold font.

Supplemental Figure 1. Distribution of the CV of protein expression at baseline. The data from the whole analyzed population (plasma, *n*=97; urine, *n*=96) were used to calculate the CV.
