# Supplementary material for: Multi-Omics Reveal Antioxidant Effects of Bardoxolone Methyl in the Phase 2 Study of Bardoxolone Methyl in Patients with CKD and Type 2 Diabetes Study
Source: Kidney360. 2025 Jun 11;6(11):1880–9. doi: 10.34067/KID.0000000853 (PMC12626677; doi:10.34067/KID.0000000853)
Supplement: Supplementary file 1 [file kidney360-6-1880-s001.pdf]

Supplemental Materials

Supplemental Table 1. Analyzed pathways in proteomics.

Pathway name	Reference
glutathione metabolic process	GO: 0006749:
glutathione biosynthetic process	GO: 0006750:
Inflammatory response pathway (*Homo sapiens*)	WikiPathways
Chemokine signaling pathway	WikiPathways
Type II interferon signaling (*H. sapiens*)	WikiPathways
Cytokines and inflammatory response (*H. sapiens*)	WikiPathways
The role of KEAP1/NRF2 pathway in skin sensitization	MetaCore
NRF2 regulation of oxidative stress response	MetaCore
Antiviral and anti-inflammatory effects of Nrf2 on SARS-CoV-2 pathway (*H. sapiens*)	WikiPathways

Supplemental Table 2. Proteins in the pathways modulated by bardoxolone methyl revealed by plasma proteomics. |Mean Log_2FC| > 0.58 are shown in bold font. Statistical analysis results with Q < 0.05 are shown in bold font. Log_2FC, log2 fold change.

Protein symbol	Protein name	Mean Log_2FC of protein expression at week 16 compared with baseline measured in plasma	Adjusted p value (Q) of Wilcoxon rank sum

		Bardoxolone methyl group	Placebo group	test of Log_2FC
NQO1	NAD(P)H quinone dehydrogenase 1	**1.498**	-0.029	< **0.0001**
TXNRD1	thioredoxin reductase 1	**1.146**	-0.073	< **0.0001**
GSTA2	glutathione S-transferase alpha 2	**1.032**	0.049	< **0.0001**
GSTA1	glutathione S-transferase alpha 1	**0.834**	0.050	< **0.0001**
GSR	glutathione-disulfide reductase	**0.831**	0.005	< **0.0001**
PRKCD	protein kinase C delta	**0.732**	-0.312	**0.020**
IDH1	isocitrate dehydrogenase (NADP(+)) 1	**0.706**	0.052	< **0.0001**
GCLM	glutamate-cysteine ligase modifier subunit	0.432	-0.138	0.14
G6PD	glucose-6-phosphate dehydrogenase	0.372	-0.019	0.36
KEAP1	kelch like ECH associated protein 1	0.316	-0.024	< **0.0001**
GSS	glutathione synthetase	0.244	-0.045	< **0.0001**
PRKCG	protein kinase C gamma	0.231	-0.024	**0.0001**
UBC	ubiquitin C	0.220	0.015	**0.0002**

CHAC2	ChaC glutathione specific gamma-glutamylcyclotransferase 2	0.190	-0.054	0.12
GPX1	glutathione peroxidase 1	0.164	-0.103	0.36
GSTZ1	glutathione S-transferase zeta 1	0.156	0.025	0.49
PRDX1	peroxiredoxin 1	0.156	-0.123	0.36
GSTO1	glutathione S-transferase omega 1	0.148	-0.089	**< 0.0001**
GSTA3	glutathione S-transferase alpha 3	0.140	0.006	0.36
TP53	tumor protein p53	0.140	-0.030	0.13
GSTM3	glutathione S-transferase mu 3	0.123	-0.021	**0.0099**
GSTM4	glutathione S-transferase mu 4	0.122	0.048	0.25
GSTM1	glutathione S-transferase mu 1	0.115	0.003	0.31
PARK7	Parkinsonism associated deglycase	0.108	-0.063	0.97
MAFG	MAF bZIP transcription factor G	0.101	-0.020	**0.0016**
MAPK3	mitogen-activated protein kinase 3	0.097	0.038	0.97

HMOX1	heme oxygenase 1	0.086	-0.007	0.12
PRKCB	protein kinase C beta	0.084	0.099	0.97
GSTT2B	glutathione S-transferase theta 2B	0.077	-0.006	0.30
RPS27A	ribosomal protein S27a	0.068	-0.114	0.89
CSNK2A1	casein kinase 2 alpha 1	0.057	-0.019	0.82
RAF1	Raf-1 proto-oncogene, serine/threonine kinase	0.057	-0.018	0.23
CUL3	cullin 3	0.056	-0.048	0.77
CREBBP	CREB binding protein	0.053	-0.039	**0.049**
MAPK1	mitogen-activated protein kinase 1	0.052	-0.004	0.86
TXN	thioredoxin	0.046	0.023	0.24
DPEP1	dipeptidase 1	0.037	0.037	0.93
GLO1	glyoxalase I	0.031	-0.060	0.95
PRKCZ	protein kinase C zeta	0.029	-0.094	0.12
PDPK1	3-phosphoinositide dependent protein kinase 1	0.019	0.046	0.96
SOD1	superoxide dismutase 1	0.017	-0.118	0.23
NFE2L2	NFE2 like bZIP transcription factor 2	0.010	0.002	0.59
GSTT2	glutathione S-transferase theta 2 (gene/pseudogene)	0.005	-0.013	0.71

GGT5	gamma-glutamyltransferase 5	0.001	0.001	0.77
PRKCI	protein kinase C iota	0.001	-0.075	0.67
CSNK2B	casein kinase 2 beta	-0.002	0.041	0.50
PIK3R1	phosphoinositide-3-kinase regulatory subunit 1	-0.003	-0.007	0.99
MAP2K1	mitogen-activated protein kinase kinase 1	-0.003	-0.022	0.84
GSTT1	glutathione S-transferase theta 1	-0.007	-0.000	0.85
GSTP1	glutathione S-transferase pi 1	-0.012	-0.053	0.81
GSK3B	glycogen synthase kinase 3 beta	-0.022	0.122	0.63
MMACHC	metabolism of cobalamin associated C	-0.024	-0.064	0.88
FYN	FYN proto-oncogene, Src family tyrosine kinase	-0.026	0.029	0.97
MAPK8	mitogen-activated protein kinase 8	-0.036	0.012	0.81
ETHE1	ETHE1 persulfide dioxygenase	-0.037	-0.016	0.95
GPX2	glutathione peroxidase 2	-0.045	0.021	0.64
CSNK2A2	casein kinase 2 alpha 2	-0.055	-0.052	0.97

GSTM5	glutathione S-transferase mu 5	-0.066	-0.008	0.85
CHAC1	ChaC glutathione specific gamma-glutamylcyclotransferase 1	-0.066	0.004	0.19
GLRX2	glutaredoxin 2	-0.072	0.007	**0.0062**
AKT2	AKT serine/threonine kinase 2	-0.072	0.084	0.33
UBB	ubiquitin B	-0.080	-0.024	0.12
PRKCQ	protein kinase C theta	-0.090	0.049	0.50
GSTA4	glutathione S-transferase alpha 4	-0.095	-0.044	0.94
GSTK1	glutathione S-transferase kappa 1	-0.098	0.126	0.33
AKT1	AKT serine/threonine kinase 1	-0.112	0.092	0.27
BACH1	BTB domain and CNC homolog 1	-0.124	-0.065	0.89
MAP2K4	mitogen-activated protein kinase kinase 4	-0.137	-0.010	**0.020**
UGT1A1	UDP glucuronosyltransferase family 1 member A1	-0.139	0.003	**< 0.0001**

HAGH	hydroxyacylglutathione hydrolase	-0.154	-0.074	0.59
SOD2	superoxide dismutase 2	-0.154	0.007	**0.0008**
PRKCA	protein kinase C alpha	-0.157	0.116	0.41
HPGDS	hematopoietic prostaglandin D synthase	-0.179	0.022	**0.0071**
ARL6IP5	ADP ribosylation factor like GTPase 6 interacting protein 5	-0.185	0.266	0.45
AKT3	AKT serine/threonine kinase 3	-0.220	0.040	**0.020**

Supplemental Table 3. Proteins in the pathways modulated by bardoxolone methyl revealed by urine proteomics. |Mean Log_2FC| > 0.58 are shown in bold font. Statistical analysis results with Q < 0.05 are shown in bold font. Log_2FC, log2 fold change.

Protein symbol	Protein name	Mean Log_2FC of protein expression at week 16 compared with baseline measured in urine		Adjusted p value (Q) of Wilcoxon rank sum test of Log_2FC
		Bardoxolone methyl group	Placebo group	

NQO1	NAD(P)H quinone dehydrogenase 1	**2.564**	0.130	< **0.0001**
TXNRD1	thioredoxin reductase 1	**2.234**	0.189	< **0.0001**
GSTA1	glutathione S-transferase alpha 1	**1.108**	0.284	0.59
GSR	glutathione-disulfide reductase	**1.002**	0.278	0.40
GSTM4	glutathione S-transferase mu 4	**0.851**	-0.019	0.11
CSF2	colony stimulating factor 2	**0.758**	0.169	0.62
CSF1	colony stimulating factor 1	**0.738**	0.135	0.057
CSF3	colony stimulating factor 3	**0.735**	0.174	0.40
GSS	glutathione synthetase	**0.716**	0.235	0.76
GCLM	glutamate-cysteine ligase modifier subunit	**0.643**	0.111	0.16
G6PD	glucose-6-phosphate dehydrogenase	0.448	0.143	0.86
PRDX1	peroxiredoxin 1	0.380	0.006	0.21
GSTM3	glutathione S-transferase mu 3	0.358	0.090	0.76
HAGH	hydroxyacylglutathione hydrolase	0.355	0.117	0.96

CHAC1	ChaC glutathione specific gamma-glutamylcyclotransferase 1	0.310	0.154	0.86
IL10	interleukin 10	0.301	0.074	0.76
IL13	interleukin 13	0.299	0.259	0.91
GSTA2	glutathione S-transferase alpha 2	0.297	-0.003	0.96
PRKCG	protein kinase C gamma	0.291	0.155	0.96
SOD2	superoxide dismutase 2	0.277	-0.215	0.37
ETHE1	ETHE1 persulfide dioxygenase	0.275	0.216	0.97
CSNK2A1	casein kinase 2 alpha 1	0.261	0.083	0.99
KEAP1	kelch like ECH associated protein 1	0.246	0.072	0.86
PRKCI	protein kinase C iota	0.238	0.159	1.0
IL1A	interleukin 1 alpha	0.233	0.119	0.87
AKT1	AKT serine/threonine kinase 1	0.189	0.149	0.99
PRKCA	protein kinase C alpha	0.167	0.075	1.0
IL2	interleukin 2	0.142	0.090	0.96
CREBBP	CREB binding protein	0.138	0.108	1.0
IL15	interleukin 15	0.137	0.099	1.0
TXN	thioredoxin	0.131	0.206	0.95

BACH1	BTB domain and CNC homolog 1	0.122	0.150	1.0
IL6	interleukin 6	0.121	0.107	0.99
DPEP1	dipeptidase 1	0.110	0.213	0.94
GSTZ1	glutathione S-transferase zeta 1	0.085	0.024	0.97
MAPK3	mitogen-activated protein kinase 3	0.084	0.094	0.95
IFNB1	interferon beta 1	0.083	0.045	0.99
MAPK1	mitogen-activated protein kinase 1	0.061	0.279	0.86
GPX1	glutathione peroxidase 1	0.058	0.225	0.70
HPGDS	hematopoietic prostaglandin D synthase	0.053	0.154	0.86
RPS27A	ribosomal protein S27a	0.039	0.069	0.99
AKT2	AKT serine/threonine kinase 2	0.021	0.057	0.96
GSK3B	glycogen synthase kinase 3 beta	0.017	-0.149	0.94
GSTA3	glutathione S-transferase alpha 3	0.015	0.400	0.77
TGFB1	transforming growth factor beta 1	0.013	0.077	0.99
GSTP1	glutathione S-transferase pi 1	-0.013	-0.053	1.0

GPX2	glutathione peroxidase 2	-0.034	0.113	0.91
UBB	ubiquitin B	-0.054	-0.013	1.0
MAP2K4	mitogen-activated protein kinase kinase 4	-0.056	0.167	0.64
MAPK8	mitogen-activated protein kinase 8	-0.067	-0.050	0.99
PARK7	Parkinsonism associated deglycase	-0.085	0.203	0.92
CSNK2B	casein kinase 2 beta	-0.088	0.059	0.81
MMACHC	metabolism of cobalamin associated C	-0.089	-0.075	0.96
CHAC2	ChaC glutathione specific gamma-glutamylcyclotransferase 2	-0.149	-0.053	0.81
HMOX1	heme oxygenase 1	-0.170	0.224	0.70
GSTM1	glutathione S-transferase mu 1	-0.172	0.005	0.73
CUL3	cullin 3	-0.239	0.071	0.70
GSTO1	glutathione S-transferase omega 1	-0.243	0.041	0.40
GLO1	glyoxalase I	-0.304	0.054	0.64
GSTT1	glutathione S-transferase theta 1	-0.331	-0.087	0.76
IL12B	interleukin 12B	-0.337	0.200	0.37

UBC	ubiquitin C	-0.345	-0.003	0.59
TP53	tumor protein p53	-0.364	0.044	0.47
PRKCB	protein kinase C beta	-0.437	-0.142	0.81
CXCL1	C-X-C motif chemokine ligand 1	-0.492	0.181	**0.031**

Supplemental Table 4. Plasma lipophilic metabolites. Statistical analysis results with Q <0.05 are shown in bold font. Log_2FC, log2 fold change.

Peak identifier	Metabolite name	Mean Log_2FC of plasma content of metabolite at week 16 compared with baseline		Adjusted p value (Q) of Wilcoxon rank sum test of Log_2FC
		Bardoxolone methyl group	Placebo group	
N_0102	LPC(18:0)	0.359	0.010	< **0.0001**
P_0037	Lactosylceramide(d18:1/18:0)	0.313	-0.019	< **0.0001**
P_0110	Cortisol	-0.044	0.050	0.71
P_0142	AEA(18:1)	-0.266	-0.148	0.2
N_0015	5-HETE	-0.686	-0.008	0.16
P_0104	Cortisone	-0.876	0.032	< **0.0001**

Supplemental Table 5. Urine water-soluble metabolites. Statistical analysis results with Q < 0.05 are shown in bold font. Log_2FC, log2 fold change.

Peak identifier	Metabolite name	Mean Log_2FC of urine content of metabolite at week 16 compared with baseline		Adjusted p value (Q) of Wilcoxon rank sum test of Log_2FC
		Bardoxolone methyl group	Placebo group	
A_0109	p-Coumaric acid; o-Coumaric acid	1.742	0.102	< **0.0001**
C_0035	Hypotaurine	1.700	0.152	< **0.0001**
C_0207	Caffeine	0.903	0.302	0.37
A_0106	N-Acetylcysteine	0.792	-0.280	**0.0052**
C_0138	His	0.622	0.088	**0.0006**
C_0306	SAM	0.562	-0.102	< **0.0001**
C_0231	Carnosine	0.499	0.169	0.54
A_0077	Tartaric acid	0.438	0.624	0.90
C_0062	Taurine	0.404	0.047	**0.016**
A_0084	4-Hydroxyphenylacetic acid; Mandelic acid; 2-Hydroxyphenylacetic acid; p-Anisic acid; Phenoxyacetic acid; 3-Hydroxyphenylacetic acid	0.310	-0.115	**0.040**
A_0254	NANA	0.260	0.004	**0.0003**

A_0082	4-Hydroxyphenylacetic acid; 3-Hydroxyphenylacetic acid	0.239	-0.186	0.12
A_0108	p-Coumaric acid; o-Coumaric acid	0.219	0.107	0.79
C_0056	Cys	0.210	-0.211	0.43
C_0058	Nicotinamide	0.199	-0.104	0.20
A_0208	3-Indoxylsulfuric acid	0.089	0.023	0.58
A_0183	Ferulic acid	0.066	0.027	0.79
A_0085	4-Hydroxyphenylacetic acid; Mandelic acid; 2-Hydroxyphenylacetic acid; p-Anisic acid; Phenoxyacetic acid; 2-(Hydroxymethyl)benzoic acid; 3-Hydroxyphenylacetic acid	-0.005	-0.117	0.79
C_0004	Methylguanidine	-0.197	-0.002	0.28
A_0117	3-(4-Hydroxyphenyl)propionic acid; 3-(3-Hydroxyphenyl)propionic acid; 2-(4-Hydroxyphenyl)propionic acid	-0.244	0.117	0.12

C_0097	1-Methylnicotinamide	-0.276	0.006	0.19
A_0110	p-Coumaric acid; o-Coumaric acid	-0.302	0.261	0.12
A_0272	Prostaglandin E2	-0.337	0.328	0.28
A_0145	Ascorbic acid	-0.439	0.238	0.47

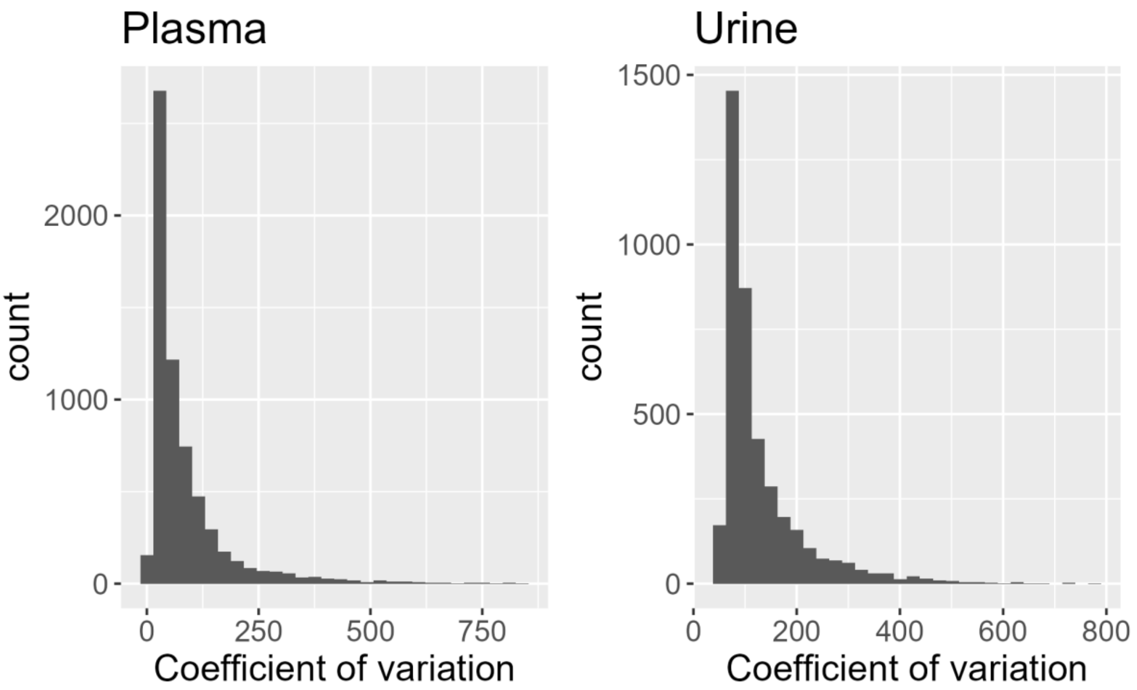

Supplemental Figure 1. Distribution of the coefficient of variation of protein expression at baseline. The data from the whole analyzed population (plasma, n = 97; urine, n = 96) were used to calculate the coefficient of variation.
