# Supplementary material for: Multi-Omics Reveal Antioxidant Effects of Bardoxolone Methyl in the Phase 2 Study of Bardoxolone Methyl in Patients with CKD and Type 2 Diabetes Study
Source: Kidney360. 2025 Jun 11;6(11):1880–9. doi: 10.34067/KID.0000000853 (PMC12626677; doi:10.34067/KID.0000000853)
Supplement: Supplementary file 2 [file kidney360-6-1880-s002.pdf]

ASN American Society of Nephrology | JASN Journal of the American Society of Nephrology | KIDNEY360 Accessing Our World From Every Angle | CJASN Clinical Journal of the American Society of Nephrology

ASN Journal Disclosure Form

As per ASN journal policy, I have disclosed any financial relationships or commitments I have held in the past 36 months as included below. I have listed my Current Employer below to indicate there is a relationship requiring disclosure. If no relationship exists, my Current Employer is not listed.

W. Haruyama reports the following:
Employer: Kyowa Kirin Co., Ltd.

I understand that the information above will be published within the journal article, if accepted, and that failure to comply and/or to accurately and completely report the potential financial conflicts of interest could lead to the following: 1) Prior to publication, article rejection, or 2) Post-publication, sanctions ranging from, but not limited to, issuing a correction, reporting the inaccurate information to the authors' institution, banning authors from submitting work to ASN journals for varying lengths of time, and/or retraction of the published work.

Name: Waka Haruyama
Manuscript ID: K360-2025-000206R1
Manuscript Title: Multi-omics reveal antioxidant effects of bardoxolone methyl in the TSUBAKI study
Date of Completion: April 13, 2025
Disclosure Updated Date: April 13, 2025

ASN
American Society of Nephrology
JASN
Journal of the American Society of Nephrology
KIDNEY360
Accessing Our World From Every Angle
CJASN
Clinical Journal of the American Society of Nephrology

ASN Journal Disclosure Form

As per ASN journal policy, I have disclosed any financial relationships or commitments I have held in the past 36 months as included below. I have listed my Current Employer below to indicate there is a relationship requiring disclosure. If no relationship exists, my Current Employer is not listed.

H. Kaneko reports the following:
Employer: Kyowa Kirin

I understand that the information above will be published within the journal article, if accepted, and that failure to comply and/or to accurately and completely report the potential financial conflicts of interest could lead to the following: 1) Prior to publication, article rejection, or 2) Post-publication, sanctions ranging from, but not limited to, issuing a correction, reporting the inaccurate information to the authors' institution, banning authors from submitting work to ASN journals for varying lengths of time, and/or retraction of the published work.

Name: Hiroto Kaneko
Manuscript ID: K360-2025-000206R1
Manuscript Title: Multi-omics reveal antioxidant effects of bardoxolone methyl in the TSUBAKI study
Date of Completion: April 11, 2025
Disclosure Updated Date: April 11, 2025

ASN American Society of Nephrology | JASN Journal of the American Society of Nephrology | KIDNEY360 Accessing Our World From Every Angle | CJASN Clinical Journal of the American Society of Nephrology

ASN Journal Disclosure Form

As per ASN journal policy, I have disclosed any financial relationships or commitments I have held in the past 36 months as included below. I have listed my Current Employer below to indicate there is a relationship requiring disclosure. If no relationship exists, my Current Employer is not listed.

T. Kitayama reports the following:
Employer: Kyowa Kirin Co., Ltd.

I understand that the information above will be published within the journal article, if accepted, and that failure to comply and/or to accurately and completely report the potential financial conflicts of interest could lead to the following: 1) Prior to publication, article rejection, or 2) Post-publication, sanctions ranging from, but not limited to, issuing a correction, reporting the inaccurate information to the authors' institution, banning authors from submitting work to ASN journals for varying lengths of time, and/or retraction of the published work.

Name: Tetsuya Kitayama
Manuscript ID: K360-2025-000206R1
Manuscript Title: Multi-omics reveal preventing resistance effects of bardoxolone methyl in the TSUBAKI study
Date of Completion: April 15, 2025
Disclosure Updated Date: April 15, 2025

ASN American Society of Nephrology
JASN Journal of the American Society of Nephrology
KIDNEY360 Accessing Our World From Every Angle
CJASN Clinical Journal of the American Society of Nephrology

ASN Journal Disclosure Form

As per ASN journal policy, I have disclosed any financial relationships or commitments I have held in the past 36 months as included below. I have listed my Current Employer below to indicate there is a relationship requiring disclosure. If no relationship exists, my Current Employer is not listed.

A. Takami reports the following:
Employer: Kyowa Kirin

I understand that the information above will be published within the journal article, if accepted, and that failure to comply and/or to accurately and completely report the potential financial conflicts of interest could lead to the following: 1) Prior to publication, article rejection, or 2) Post-publication, sanctions ranging from, but not limited to, issuing a correction, reporting the inaccurate information to the authors' institution, banning authors from submitting work to ASN journals for varying lengths of time, and/or retraction of the published work.

Name: Atsuko Takami
Manuscript ID: K360-2025-000206R1
Manuscript Title: Multi-omics reveal antioxidant effects of bardoxolone methyl in the TSUBAKI study
Date of Completion: April 14, 2025
Disclosure Updated Date: April 14, 2025

ASN American Society of Nephrology | JASN Journal of the American Society of Nephrology | KIDNEY360 Accessing Our World From Every Angle | CJASN Clinical Journal of the American Society of Nephrology

ASN Journal Disclosure Form

As per ASN journal policy, I have disclosed any financial relationships or commitments I have held in the past 36 months as included below. I have listed my Current Employer below to indicate there is a relationship requiring disclosure. If no relationship exists, my Current Employer is not listed.

T. Tomiyama reports the following:
Employer: Kyowa Kirin; and Ownership Interest: AEON Co., Ltd.; Japan Post Holdings Co., Ltd.;.

I understand that the information above will be published within the journal article, if accepted, and that failure to comply and/or to accurately and completely report the potential financial conflicts of interest could lead to the following: 1) Prior to publication, article rejection, or 2) Post-publication, sanctions ranging from, but not limited to, issuing a correction, reporting the inaccurate information to the authors' institution, banning authors from submitting work to ASN journals for varying lengths of time, and/or retraction of the published work.

Name: Tetsuro Tomiyama
Manuscript ID: K360-2025-000206R1
Manuscript Title: Multi-omics reveal antioxidant effects of bardoxolone methyl in the TSUBAKI study
Date of Completion: April 14, 2025
Disclosure Updated Date: April 14, 2025

ASN
American Society of Nephrology
JASN
Journal of the American Society of Nephrology
KIDNEY360
Accessing Our World From Every Angle
CJASN
Clinical Journal of the American Society of Nephrology

ASN Journal Disclosure Form

As per ASN journal policy, I have disclosed any financial relationships or commitments I have held in the past 36 months as included below. I have listed my Current Employer below to indicate there is a relationship requiring disclosure. If no relationship exists, my Current Employer is not listed.

K. Yamasaki reports the following:
Employer: Kyowa Kirin. Co Ltd; Ownership Interest: Kyowa Kirin. Co Ltd; and Research Funding: Kyowa Kirin. Co Ltd.

I understand that the information above will be published within the journal article, if accepted, and that failure to comply and/or to accurately and completely report the potential financial conflicts of interest could lead to the following: 1) Prior to publication, article rejection, or 2) Post-publication, sanctions ranging from, but not limited to, issuing a correction, reporting the inaccurate information to the authors' institution, banning authors from submitting work to ASN journals for varying lengths of time, and/or retraction of the published work.

Name: Kohei Yamasaki
Manuscript ID: K360-2025-000206R1
Manuscript Title: Multi-omics reveal antioxidant effects of bardoxolone methyl in the TSUBAKI study.
Date of Completion: April 15, 2025
Disclosure Updated Date: April 15, 2025

ASN American Society of Nephrology | JASN Journal of the American Society of Nephrology | KIDNEY360 Accessing Our World From Every Angle | CJASN Clinical Journal of the American Society of Nephrology

ASN Journal Disclosure Form

As per ASN journal policy, I have disclosed any financial relationships or commitments I have held in the past 36 months as included below. I have listed my Current Employer below to indicate there is a relationship requiring disclosure. If no relationship exists, my Current Employer is not listed.

K. Yoshioka reports the following:
Employer: Kyowa Kirin.Co, Ltd.; and Ownership Interest: Kyowa Kirin.Co, Ltd.

I understand that the information above will be published within the journal article, if accepted, and that failure to comply and/or to accurately and completely report the potential financial conflicts of interest could lead to the following: 1) Prior to publication, article rejection, or 2) Post-publication, sanctions ranging from, but not limited to, issuing a correction, reporting the inaccurate information to the authors' institution, banning authors from submitting work to ASN journals for varying lengths of time, and/or retraction of the published work.

Name: Kentaro Yoshioka
Manuscript ID: K360-2025-000206R1
Manuscript Title: Multi-omics reveal antioxidant effects of bardoxolone methyl in the TSUBAKI study
Date of Completion: April 13, 2025
Disclosure Updated Date: April 13, 2025
